# Ablation of TRPM5 in Mice Results in Reduced Body Weight Gain and Improved Glucose Tolerance and Protects from Excessive Consumption of Sweet Palatable Food when Fed High Caloric Diets

**DOI:** 10.1371/journal.pone.0138373

**Published:** 2015-09-23

**Authors:** Marie H. Larsson, Pernilla Håkansson, Frank P. Jansen, Kerstin Magnell, Peter Brodin

**Affiliations:** 1 Department of Bioscience, Cardiovascular & Metabolic Disease Innovative Medicines, AstraZeneca R&D, Mölndal, Sweden; 2 Department of Discovery Safety, Drug Safety and Metabolism, AstraZeneca R&D, Mölndal, Sweden; 3 Department of Screening Sciences & Compound Management, Discovery Sciences, AstraZeneca R&D, Mölndal, Sweden; 4 Department of Reagents and Assay Development, Transgenics, Discovery Sciences, AstraZeneca R&D, Mölndal, Sweden; Sapienza University of Rome, ITALY

## Abstract

The calcium activated cation channel transient receptor potential channel type M5 (TRPM5) is part of the downstream machinery of the taste receptors and have been shown to play a central role in taste signalling. In addition it is also found in other types of chemosensory cells in various parts of the body as well as in pancreatic β-cells. The aim of this study was to investigate the effects of TRPM5 gene ablation on body weight, insulin sensitivity and other metabolic parameters in long-term high caloric diet induced obesity. *Trpm5* ^*-/-*^ mice gained significantly less body weight and fat mass on both palatable carbohydrate and fat rich cafeteria diet and 60% high fat diet (HFD) and developed less insulin resistance compared to wild type mice. A main finding was the clearly improved glucose tolerance in *Trpm5* ^***-/-***^ mice compared to wild type mice on cafeteria diet, which was independent of body weight. In addition, it was shown that *Trpm5* ^*-/-*^ mice consumed the same amount of calories when fed a HFD only or a HFD in combination with a palatable chocolate ball, which is in contrast to wild type mice that increased their caloric intake when fed the combination, mainly due to excessive consumption of the chocolate ball. Thus the palatable sugar containing diet induced overeating was prevented in *Trpm5* ^*-/-*^ mice. This indicates that sweet taste induced overeating may be a cause for the increased energy intake and glucose intolerance development seen for wild type mice on a sugar and high fat rich cafeteria diet compared to when on a high fat diet. This study point to an important role for the taste signalling system and TRPM5 in diet induced glucose intolerance.

## Introduction

The past decades have witnessed an epidemic in obesity, which is closely paralleled by an increase of type 2 diabetes [1,2]. Several studies show that weight gain and obesity are associated with an increased risk of developing diabetes [3,4], thus there is a strong relationship between obesity and type 2 diabetes and there is a widespread concern that the abundance of sugar- and fat-rich food is contributing to the obesity epidemic [5–9]. Rodent studies have shown that unlimited access to high caloric diets leads to diet induced obesity and reduced insulin sensitivity and that diet induced obesity is due to several oral- (taste and texture) and post-oral (nutrient, energy content and activation of reward circuits) factors [10,11], as well as reduced energy expenditure.

Taste receptors such as T1R2/R3 (sweet receptor) and their downstream signalling machinery have attracted considerable attention in obesity and metabolism research not only because they contribute to oral evaluation of food by virtue of their expression in taste buds [12–14], but also since they are present in enteroendocrine cells, thereby potentially contributing to post oral ingestive control and glucose homeostasis [15–17]. Taste receptors couple to the G-protein α-gustducin, leading to elevation of intracellular calcium. This opens the calcium activated cation channel, transient receptor potential channel type M5 (TRPM5) [18–20], which depolarizes the cell triggering neuronal responses to the taste centers and possibly also leads to secretion of GLP-1 and other hormones from enteroendocrine cells where the involvement of taste receptors have been suggested [21]. While this is not the only mechanism by which glucose depolarises enteroendocrine cells, it is nevertheless a candidate for therapeutic intervention. While TRPM5 has a central role in taste signalling, being critical for sweet, bitter and umami taste perception [22,23], the *Trpm5* gene is also expressed in other types of chemosensory cells in various parts of the body [24–27], as well as in pancreatic β-cells [28]. The overall mRNA expression in tissues is low, and the protein levels are unknown, as no reliable TRPM5 antibody has been developed. Deletion of the *Trpm5* gene has been shown to reduce insulin secretion in mice [29,30] and insulin has been shown to downregulate expression of TRPM5 in pancreatic β-cells [31]. In addition, TRPM5 has been shown to be important for preference of fat taste [32,33], as well as non-sweet tasting carbohydrates such as maltodextrin [32] and starch [34]. TRPM5 has also been shown to be important for the release of endogenous Opioids from the duodenum [35].

While the taste deficits in different strains of *Trpm5* ^*-/-*^ mice are well documented [22,23] and the effect on acute insulin secretion from pancreatic islets have been demonstrated [29,30], very little is known about the effect of TRPM5 ablation on other metabolic parameters. It has been shown that *Trpm5* ^*-/-*^ mice have a reduced consumption of carbohydrate rich liquid diets and reduced weight gain [36] over short term, but no studies have been published examining the effect of TRPM5 ablation on metabolic parameters after long term high fat/high sugar or high fat diet feeding.

The aim of the present study was to investigate the role of TRPM5 gene ablation on body weight, insulin sensitivity and other metabolic parameters in long-term high caloric diet induced obesity. In brief, we have showed that *Trpm5* ^*-/-*^ mice gained significantly less body weight and fat mass on both palatable carbohydrate rich cafeteria diet and 60% HFD and were more glucose tolerant compared to wild type mice, which after cafeteria diet induced obesity was largely independent of body weight. We also demonstrated that *Trpm5* ^*-/-*^ mice were protected from palatable sugar containing diet induced overeating.

## Research Design and Methods

### Ethical statements

All the experiments were approved by the Animal Ethics Review Committee in Göteborg, Sweden and carried out in strict accordance to recommendations for the care and use of laboratory animals.

### Animals

The *Trpm5* ^*-/-*^ strain B6.129P2-*Trpm5* ^*tm1Dgen*^/J, where the *Trpm5* ^*-/-*^ gene is targeted by an insertion of LacZ in exon 16, was originally created by Deltagen Inc. (San Carlos, CA). Cryopreserved embryos were purchased from The Jackson Laboratory (Stock # 5848, Bar Harbor, ME) where the *Trpm5* ^*-/-*^ mice had been backcrossed for at least 7 generations towards the C57BL/6J genetic background. The mouse strain was further backcrossed with C57BL/6J mice and bred in-house. Littermate mice were used as controls in the studies. Gene targeting was confirmed in our laboratory by PCR and at transcriptional level in several tissues by RTPCR (data not shown). General phenotyping results (which did not show any apparent phenotype in unchallenged lean animals) and genotyping protocol can be found at the Jackson Lab JAXmice web page.

The mice were group housed (unless otherwise described) within each genotype and had free access to food and tap water. The cages were kept in a temperature-controlled environment (20–21°C) with a relative humidity of 40–60% and a light-dark cycle of 12 h (lights on at 06:00 and lights off at 18:00). Littermate *Trpm5* ^*-/-*^ and *Trpm5* ^*+/+-*^ (wild type) control mice used in all studies were individually marked with a tattoo on the tail.

The first batch of experimental mice generated 10 female and 4 male *Trpm5* ^*-/-*^ and 16 female and 4 male wild type control mice. The male mice were used in the preference tests to verify that the mice were functionally deficient of sweet taste. The female mice were used for the long term high caloric diet study where the mice in addition to regular mouse chow were given a high energy fat and sugar containing cafeteria diet, or regular mouse chow only.

The second batch of experimental mice was used for a long term high caloric diet study where the mice were given a high fat diet (HFD) only. Only the female mice were used (15 *Trpm5* ^*-/-*^ and 13 wild type control).

The third batch of experimental mice were used for acute food intake measurements in female mice (12 *Trpm5* ^*-/-*^ and 12 wild type) and effect on body weight gain and energy expenditure in male mice (6 *Trpm5* ^*-/-*^ and 8 wild type).

### Two bottle preference test (lickometer test)

The lickometer system used for the two bottle preference test was constructed in a similar way as reported previously [37], utilizing electrical sensors. Briefly, the system consisted of regular plastic cages (8 cages) with one half of the cage covered by a metal plate, while the other half was covered with aspen wood shavings and contained bedding and nesting material. Two 250ml water bottles per cage were placed at the metal plated side and copper wires were wrapped around the water sippers and isolated. The wires were then connected to amplifiers and analogue/digital converters and the grounds were connected to the metal plates. Each lick closes the electrical circuit for the duration of tongue-sipper contact, which results in a very small current (0.25 μA). A lick was defined when the circuit was closed and resulted in an amplitude of at least 2 V with a frequency of at least 35 ms.

### Acute preference test

To test the acute preference for sucrose (0 (water), 0.1, 0.3 and 1 M) and sucralose (0 (water), 1, 10 and 30 mM), *Trpm5* ^*-/-*^ and wild type mice were single housed in the lickometer system, with free access to food and liquid. Recordings of the number of licks were performed during 16 h, starting 3 h before the dark period. A test session consisted of 4 days, in the first two days the mice received water in both bottles (baseline), thereafter they received water in one bottle and test solution in the other. The position of the test solution was switched between day 3 and day 4, in order to control for side preference. The mice were then group housed in their home cages for 3 days before a new test session started. During four consecutive test sessions (4 weeks), mice were randomly assigned to receive one concentration of the test solution each week (starting with sucrose and followed by sucralose at the next 4 weeks sessions). The average number of licks of each solution concentration was calculated and compared with water over the four weeks test session.

### Nutrient content preference test

A two week lickometer experiment was performed to test the ability of the mice to sense and show preference for the nutrient content of the liquids. During the first week (baseline), the mice received water in both bottles. On the second week, one bottle contained test solution, and one bottled contained water, with the position of the test solution switched every day. The concentrations of the test solutions were 1 M Sucrose or 10 mM Sucralose, since these concentrations elicited the highest response in the acute response test. Total number of licks and a preference score (percent licks on the test solution of the total number of licks) were calculated for each mouse for each day. The average preference score for all mice with respective genotype was calculated for each day.

### Long term high caloric diet studies

**Fig 1:**
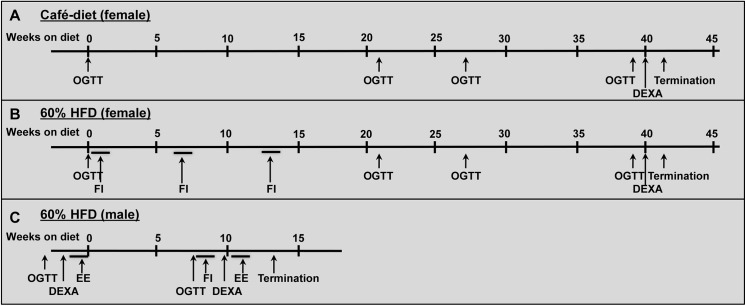
Schematic presentation of the experimental protocols used in the long term diet studies. From 12 weeks of age female *Trpm5* ^*-/-*^ and wild type mice were fed normal mouse chow together with a cafeteria diet (high fat-high sugar) (n = 5-7/strain) (A) or a 60% HFD only (n = 13-15/strain) (B). The mice were weighed weekly, oral glucose tolerance tests (OGTTs) were performed at the start of the study and at different time-points during the studies and a DEXA scan was run at the end of the studies. In the female 60% HFD study, food intake was recorded for 4 days at 3 different occasions. C; Male *Trpm5* ^*-/-*^ and wild type mice were fed 60% HFD (n = 8/strain) from the age of 15 weeks.The mice were weighed weekly and energy expenditure, OGTT and DEXA scan were performed before and after the diet treatment. FI = Food Intake, EE = Energy Expenditure.

Two separate long-term high caloric diet studies were performed using either cafeteria diet or HFD in female mice (4–5 mice/cage), starting at an age of 12 weeks. In the first high caloric diet study female *Trpm5* ^-/-^ and wild type mice were given, in addition to regular mouse chow (R3, Lactamine AB, Stockholm, Sweden, 3.0 Kcal/g), a high energy fat and sugar containing cafeteria diet consisting of nougat (Odense Marcipanfabrik, Odense, Denmark, 5.6 Kcal/g), chocolate ball (Delicatoboll, Delicato Bakverk AB, Huddinge, Sweden, 5.0 Kcal/g), chocolate (Marabou milk chocolate, Marabou Sweden, 5.4 Kcal/g) and cheese (Västervik Gräddost, Arla Foods, Sweden, 4.2 Kcal/g) (Table 1) for 40 weeks. In the second high caloric diet study, female *Trpm5* ^*-/-*^ and wild type mice were given a HFD containing (energy percentage) 60% fat, 20% carbohydrates, and 20% protein (D12492; Research Diets, New Brunswick, NJ, USA, 5.2 Kcal/g, Table 1) for 40 weeks. In both studies, the mice were weighed weekly, an oral glucose tolerance test (OGTT) was performed at the start of the studies and at different time-points during the studies and body composition analysis was performed at the end of the studies (Fig 1). At the day of termination (9–11 a.m.), plasma was isolated from isoflurane anesthetized mice and organs were collected, weighed and snap frozen in liquid N_2_ and stored at -80°C for further analysis of metabolic parameters.

**Table 1:** Detailed composition of the diets used.

	Energy content	Fat	Carbohydrate	Protein	Total
Chocolate Ball	Kcal/g	2.73	2.12	0.2	5.05
Chocolate Ball	Kcal %	54	42	4	100
Nougat	Kcal/g	3.44	1.88	0.24	5.56
Nougat	Kcal %	62	32	4	100
Marabou milk chocolate	Kcal/g	2.82	2.32	0.24	5.38
Marabou milk chocolate	Kcal %	52	43	5	100
Cheese	Kcal/g	3.35	0.04	0.8	4.19
Cheese	Kcal %	80	1	19	100
HFD	Kcal/g	1.83	1.37	1.37	5.24
HFD	Kcal %	60	20	20	100

The Chocolate ball is made of; sugar, oatmeal, vegetable fat, cacao powder, salt, aroma of mocha and vanillin and preservatives and there are coconut flakes on top of them.

In the female HFD study, food intake was grossly recorded by weighing the cage metal mesh lid every day for 4 days during the first, 6^th^ and 13^th^ week of the study (Fig 1B). The total amount of food eaten for one cage was divided by the number of mice in the cage and presented as average food intake/ mouse/ day.

A third high caloric study were performed where male *Trpm5* ^*-/-*^ and wild type mice (starting at an age of 15 weeks) were given a HFD (D12492; Research Diets, New Brunswick, NJ, USA) for 12 weeks in order to compare gender differences on body weight gain and study effects on energy expenditure. In the male HFD study, energy expenditure and body composition analysis was performed before the start and at the end of the study (Fig 1C).

### Body composition

Body composition analysis was performed on anesthetised (Forene, Abbot, Sweden) mice by dual-energy X-ray absorptiometry (DEXA) (PIXImus Lunar, GE Medical Systems, Madison, WI).

### Oral glucose tolerance test (OGTT) and HOMA-IR index

The mice were fasted for 4 h in the beginning of their light period. A blood sample was taken from the tail vein at 0 (before) and 15, 30, 60 & 120 min after oral glucose administration (2 g/kg, 6.67 ml/kg, oral gavage using a feeding needle of stainless steel from AgnTho´s, Sweden), and glucose (~2 μl whole blood, AccuChek^®^;Roche Diagnostics; Mannheim, Germany) and insulin (2 x 3 μl whole blood, Ultra sensitive mouse insulin ELISA Kit, Chrystal Chem INC, Downers Grove, IL, USA) levels were analyzed. Homeostatic model assessment of insulin resistance (HOMA-IR) index was calculated as (Insulin (μU/ml) x glucose (mM))/22.5.

### Indirect calorimetry and spontaneous locomotor activity

Oxygen consumption (vO_2_) and carbon dioxide production (vCO_2_) were measured using a CLAMS open circuit calorimetry system (Oxymax, Columbus Instruments, Columbus, OH, USA). The mice were placed in calorimeter chambers with ad libitum access to water and regular mouse chow (before the HFD study start) or HFD for 72 h. Energy expenditure (Kcal/h) was calculated: (3.815 + 1.232 RER) × vO_2_, where RER is the respiratory exchange ratio [volume of CO_2_ produced per volume of O_2_ consumed (both ml/kg/min)] and vO_2_ is the volume of O_2_ consumed per h per kg mass of the animal. The value of energy expenditure was correlated to individual lean body mass. Horizontal spontaneous locomotor activity was measured continuously in the CLAMS system. Data analysis was performed using CLAMS (Columbus Instruments) and Spotfire (TIBCO, MA, USA) software.

### Acute food intake measurements

Food intake was recorded using an in-house built automatic food intake analysis system (AstraZeneca R&D, Mölndal, Sweden) consisting of two food hoppers (independently connected to a scale) which continuously register food intake. A feeding bout was defined as continuously feeding for more than 5 s, a minimum food intake (bout weight) of 10 mg and a relapse time of 5 s between the bouts. The food intake was binned into hours and cumulative food intake per hour was calculated. All data was collected using Microsoft Excel and analysis was performed using Excel and Spotfire (TIBCO, MA, USA) software.

Group housed female *Trpm5* ^*-/-*^ and wild type mice (19–20 weeks old) were switched from regular chow to HFD (D12492, 5.2 Kcal/g) and a fat/sugar rich, palatable chocolate ball containing (energy percentage) 54% fat, 42% carbohydrates, and 4% protein (Delicatoboll, Delicato Bakverk AB, Huddinge, Sweden, 5.0 Kcal/g) one week before they were placed in the food intake analysis system, where they were singled housed with free access to two food hoppers and water. Food intake, HFD in one food hopper and a chocolate ball in the other, was automatically recorded for 11 consecutive days. The mice were group housed and fed regular chow for 4 weeks before initiating a second food intake analysis study exploring food intake when only given the HFD. The cages were checked for spillage and new fresh HFD pellets were added every day, while the chocolate ball was changed after 7 days. Body weights were measured every second day. The 24h cumulative food intake was calculated for each day and then averaged over 11 days. The total energy intake and the preference for HFD versus the chocolate ball were calculated.

### Statistics

All data are expressed as mean ± SEM. Statistical significance for body weight gain was analysed in a repeated measures mixed effect model with baseline weight as covariate and an interaction term between genotype and time included, to allow for different behaviour over time. The correlation structure between the different measurements within animal was taken to be AR (1) (auto-regressive: the correlation between successive measurements decrease with increasing difference in time between the measurements). A separate analysis was performed for each diet.

ANOVA with Bonferroni post-hoc test was used in cases where two or more comparisons were performed and Student’s unpaired t-test was used in all other studies and comparisons performed, using GraphPad Prism 4 software (San Diego, CA, USA). Data was considered statistically significant when P<0.05.

## Results

### *Trpm5* ^*-/-*^ mice lack sweet taste preference

**Fig 2:**
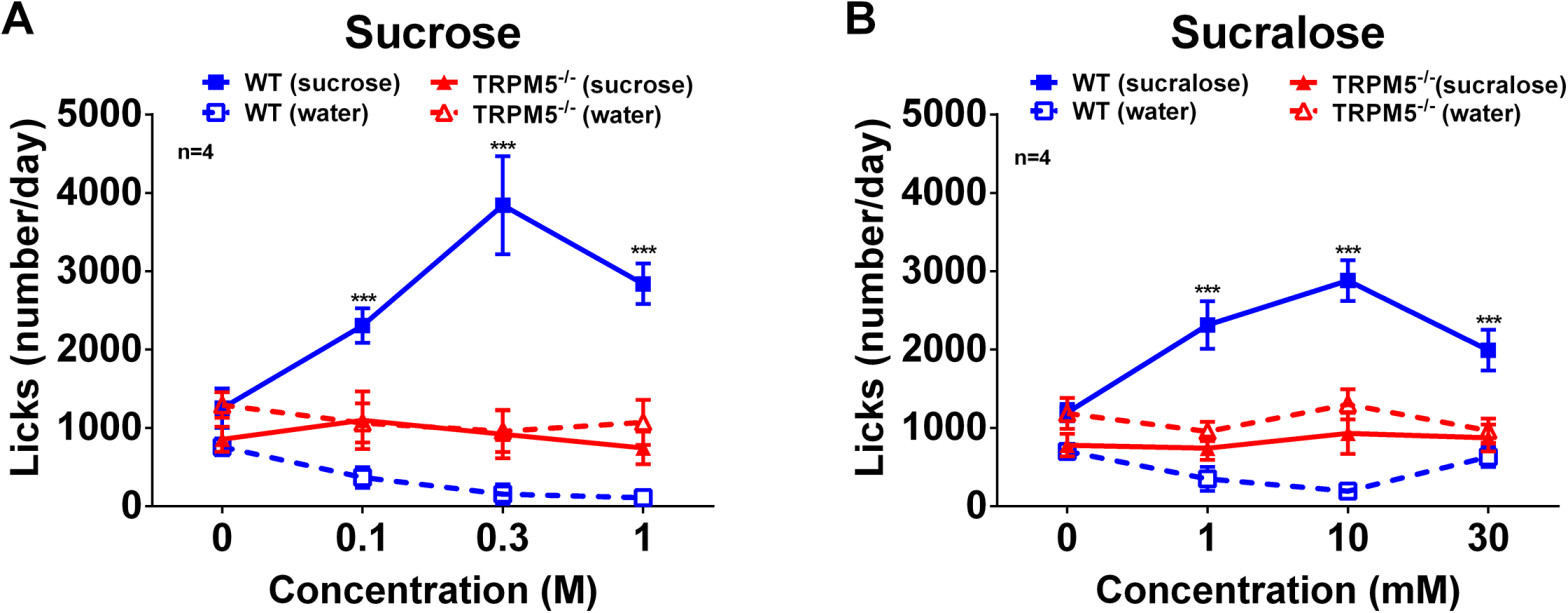
*Trpm5* ^*-/-*^ mice do not have any acute preference for sweet taste. Acute two bottle taste preference test in male *Trpm5* ^*-/-*^ and wild type mice under conditions of free access to water in one bottle and sweetener in the other. Average daily (16h, starting 3h before active period) number of licks of water and sucrose (A) or sucralose (B) for *Trpm5* ^*-/-*^ and wild type mice. Data are presented as mean ± SEM, n = 4, *** p<0.001, water vs sweetener within the same genotype (Students t-test).

Male *Trpm5* ^*-/-*^ mice did not show any acute preference for either sucrose or the artificial sweetener sucralose in a two-bottle lickometer test. In contrast, wild type littermate controls showed a concentration dependent preference for both sucrose and sucralose (Fig 2). This clearly demonstrates that the *Trpm5* ^*-/-*^ mice are functionally deficient in their sweet taste perception.

### *Trpm5* ^*-/-*^ and wild type mice show similar phenotypes before starting on high caloric diets

**Fig 3:**
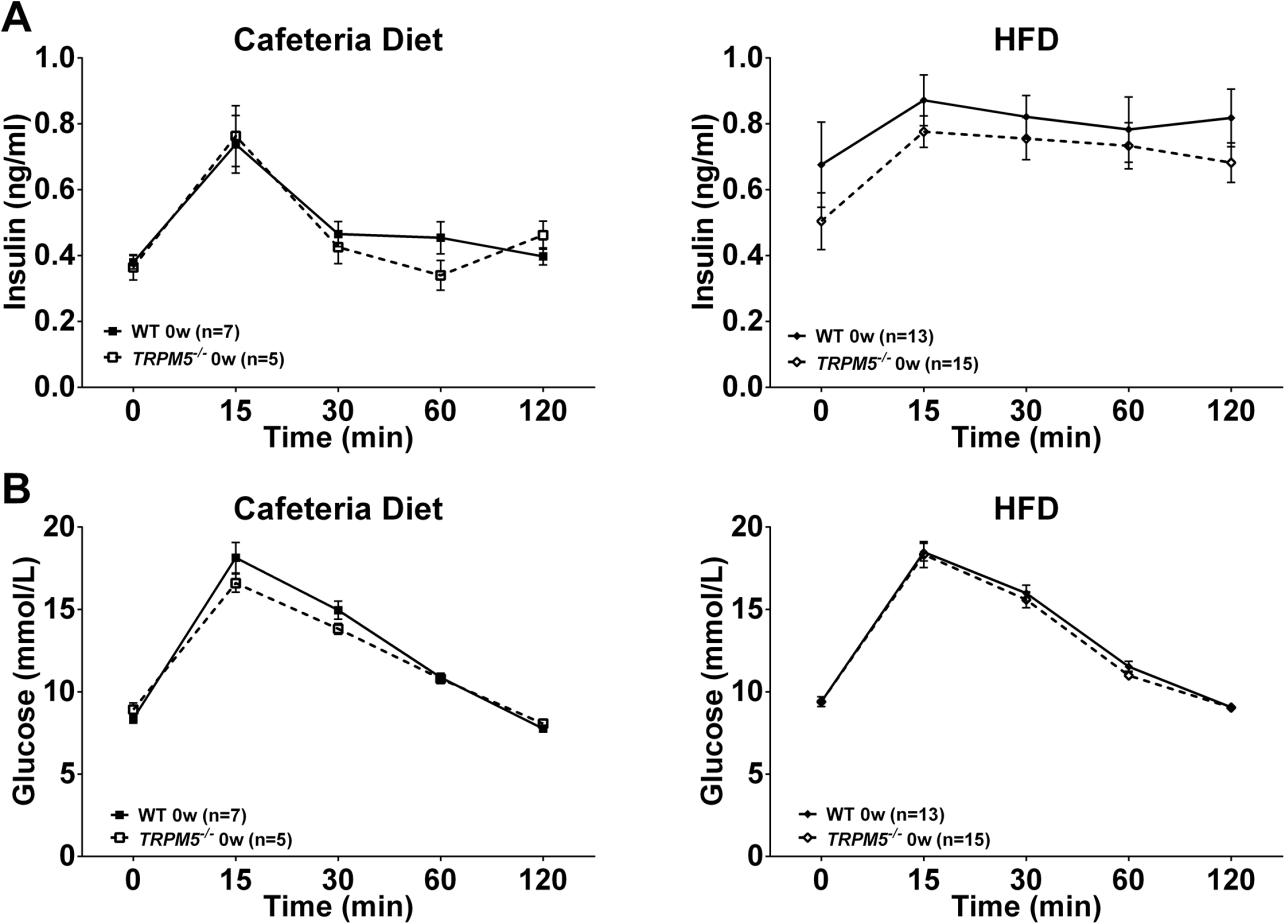
Effects of phenotype on glucose and insulin responses after an oral glucose tolerance test in female *Trpm5* ^*-/-*^ and wild type mice. At the age of 12 weeks, before starting on a diet, an oral glucose tolerance test was performed after a 4h fasting and (A) insulin and (B) glucose levels were recorded. Data are presented as mean ± SEM.

At the age of 12 weeks, before starting on a high caloric diet, female *Trpm5* ^*-/-*^ and wild type mice had similar body weights (regular chow diet, 21.1 ± 0.3 vs 20.4 ± 0.9; cafeteria diet; 20.9 ± 0.5 vs 21.3 ± 0.5 and HFD 22.4 ± 0.4 vs 21.1 ± 0.2, *Trpm5* ^*-/-*^ and wild type mice respectively). They also showed similar glucose and insulin levels before (fasting) and after an oral glucose challenge (oral glucose tolerance test, OGTT) (Fig 3).

### *Trpm5* ^*-/-*^ mice show attenuated high caloric diet-induced weight gain

**Fig 4:**
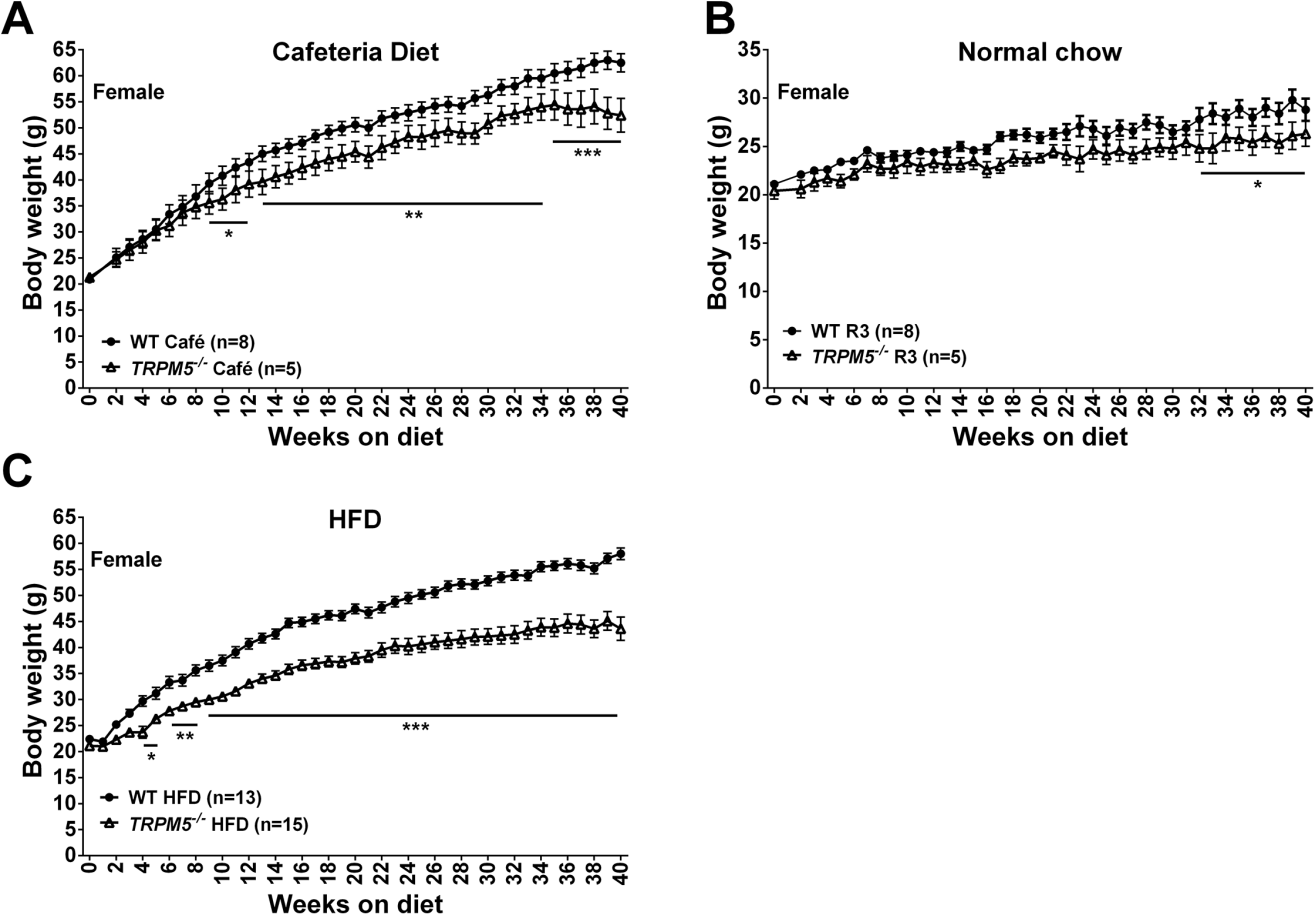
Effects of diet on body weight gain in *Trpm5* ^*-/-*^ and wild type mice. At the age of 12 weeks Female *Trpm5* ^*-/-*^ and wild type mice were fed normal mouse chow (R3) (A) or in addition to normal mouse chow (R3) offered a cafeteria diet (B) or switched to a 60% HFD (C), and body weight was recorded weekly. Data are presented as mean ± SEM, *p<0.05, **p<0.01, ***p<0.001, *Trpm5* ^*-/-*^ vs wild type at the same diet. (Statistics, see Material and Methods).

When fed the cafeteria diet, female *Trpm5* ^*-/-*^ mice gained significantly less body weight (16% after 40 weeks, p<0.001, Fig 4A), had significantly less total fat and lean body mass, liver and brown adipose tissue (BAT) weights and reduced liver triglyceride content compared to wild type littermate controls after being 40 weeks on the diet (Table 2). Interestingly, also when fed a regular chow diet, female *Trpm5* ^*-/-*^ mice gained significantly less body weight (9%, p<0.05, Fig 4B) and had reduced liver weights and liver triglyceride content (Table 2) compared to wild type mice.

**Table 2:** The effects of cafeteria diet and HFD on body composition, liver weight, adipose tissue and plasma biomarkers in female *Trpm5* ^*-/-*^ (KO) and wild type (WT) mice at the time of necropsy.

	Normal Chow (R3)		Cafeteria diet		HFD	
	WT (n = 8)	KO (n = 5)	WT (n = 8)	KO (n = 5)	WT (n = 10)	KO (n = 12)
Body weight (g)	28.8 ± 1.1	26.3 ± 1.3^a^	62.5 ± 1.7	52.4 ± 3.2^a^	58.0 ± 1.1	43.6 ± 2.2^b^
Total fat mass (g)	7.31 ± 0.79	5.68 ± 0.80	37.5 ± 1.5	29.5 ± 2.4^a^	34.9 ± 0.9	23.9 ± 1.9^c^
Lean tissue mass (g)	21.3 ± 0.5	20.1 ± 0.6	26.4 ± 0.7	23.3 ± 0.9^a^	21.3 ± 0.2	18.4 ± 0.3^c^
Total fat (% of BW)	25.0 ± 1.8	21.7 ± 2.1	58.6 ± 1.0	55.5 ± 1.5	62.0 ± 0.5	55.6 ± 1.7^b^
Ovary fat (% of BW)	3.74 ± 0.49	3.30 ± 0.55	11.1 ± 0.5	11.4 ± 0.5	11.0 ± 0.5	8.6 ± 0.5^c^
BAT (% of BW)	0.29 ± 0.02	0.26 ± 0.03	0.83 ± 0.08	0.43 ± 0.06^b^	0.61 ± 0.06	0.39 ± 0.04^b^
Liver weight (g)	1.22 ± 0.05	0.88 ± 0.10^b^	3.90 ± 0.28	2.25 ± 0.47^b^	1.56 ± 0.10	1.08 ± 0.05^c^
Liver weight (% of BW)	4.16 ± 0.15	3.39 ± 0.31^a^	5.96 ± 0.31	3.90 ± 0.56^b^	2.67 ± 0.13	2.53 ± 0.08
Liver TG (g/100 g liver)	4.50 ± 0.57	2.48 ± 0.29^a^	18.9 ± 1.6	16.1 ± 2.5	17.2 ± 1.9	8.01 ± 1.01^c^
Plasma TG (mM)	0.69 ± 0.09	0.58 ± 0.08	0.40 ± 0.07	0.42 ± 0.05	0.39 ± 0.06	0.33 ± 0.02
Leptin (ng/ml)	8.15 ± 1.67	4.86 ± 1.01	73.9 ± 0.3	72.5 ± 2.3	134.9 ± 6.6	86.4 ± 10.2^c^

Body weight and total fat and lean tissue mass was analysed (DEXA) after being on respective diet for 40 weeks (52 weeks old). One week later plasma was isolated from isoflurane anesthetized mice and organs were collected, weighed and snap frozen in liquid N_2_ and stored at -80°C. Data are presented as mean ± SEM.

^a^p<0.05

^b^p<0.01

^c^p<0.001 *Trpm5* ^*-/-*^ vs corresponding wild type control within respective diet (students t-test).

The difference in weight gain between female *Trpm5* ^*-/-*^ and wild type mice on cafeteria diet could be due to the lack of sweet taste perception in the *Trpm5* ^*-/-*^ mice leading to decreased intake of the sweet and highly palatable cafeteria diet. Therefore, a second long-term body weight study was performed using a HFD. Again, the female *Trpm5* ^*-/-*^ mice gained significantly less body weight (25% after 40 weeks, p<0.001, Fig 4C), had significantly less total fat and lean body mass, ovarian fat pad weight, brown adipose tissue weight, liver triglyceride content and circulating leptin levels compared to wild type littermate controls (Table 2). There was no apparent difference in food intake between female *Trpm5* ^*-/-*^ and wild type mice at any of the time points recorded (1^st^, 6^th^ and 13^th^ week of the study, data not shown) which potentially could have explained the difference in weight gain. Also the male *Trpm5* ^*-/-*^ mice gained significantly less body weight compared to wild type littermate controls. After 12 weeks on HFD they had gained 17% less body weight compared to wild type littermate controls (data not shown).

### *Trpm5* ^*-/-*^ mice are resistant to high caloric diet-induced glucose intolerance

**Fig 5:**
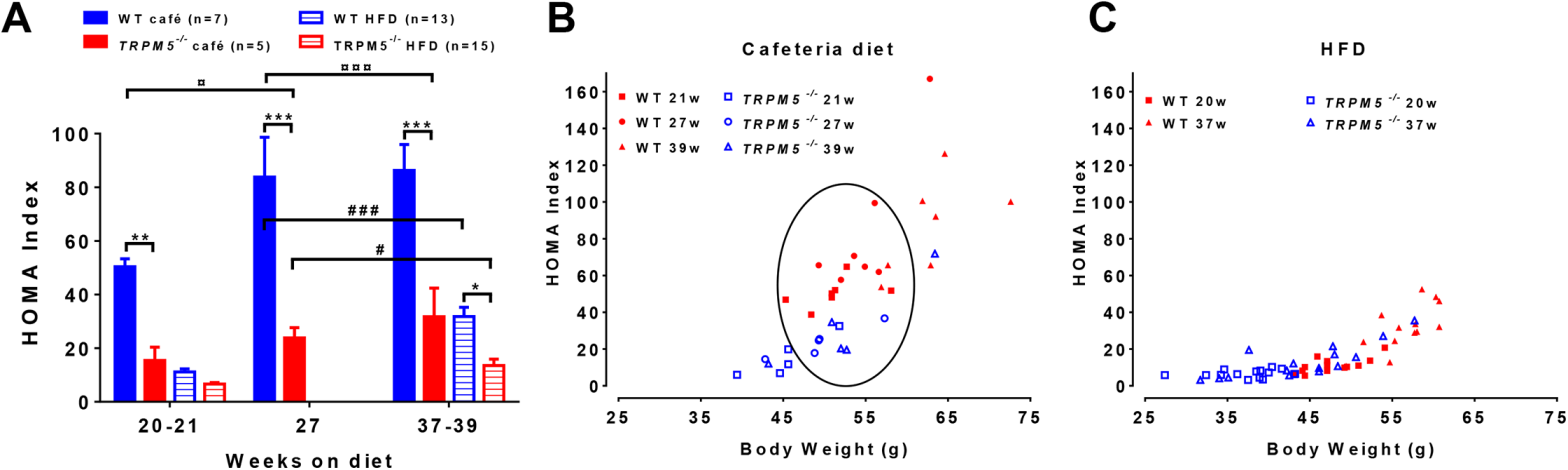
Effects of diet on basal insulin and glucose levels in female *Trpm5* ^*-/-*^ and wild type mice. Baseline glucose and insulin levels were recorded after a 4h fasting at different time points (weeks) after start on diet. (A) The group HOMA index was calculated. Individual HOMA index were then related to individual bodyweight for cafeteria diet (B) and HFD (C). The circle emphasize where *Trpm5* ^*-/-*^ and wild type mice have similar body weight, but *Trpm5* ^*-/-*^ have a reduced HOMA:IR vs wild type. Data are presented as mean ± SEM, **p<0.01, ***p<0.001 T*rpm5* ^*-/-*^ vs wild type at corresponding age and diet, ^¤^p<0.05, ^¤¤¤^p<0.001,*Trpm5* ^*-/-*^ vs wild type at corresponding bodyweight and diet, ^#^p<0.05, ^###^p<0.001 cafeteria diet vs HFD for respective genotype (ANOVA with Bonferroni post-hoc test).

Female *Trpm5* ^*-/-*^ mice compared to age matched wild type mice showed reduced fasting glucose, insulin levels and HOMA-IR index when fed either cafeteria diet or HFD (Table 3, Fig 5A). This could be due to the lower body weight of the *Trpm5* ^*-/-*^ mice when compared to age matched wild type mice. However, when comparing cafeteria diet fed female *Trpm5* ^*-/-*^ mice with weight matched younger wild type mice, *Trpm5* ^*-/-*^ mice still exhibited lower fasting glucose and insulin levels as well as significantly improved HOMA-IR index, i.e. the HOMA-IR index was reduced with 57–62% (p<0.05) (Table 3, Fig 5A and 5B). Thus, indicating that *Trpm5* ablation improves glucose homeostasis independent of the resistance to cafeteria diet-induced body weight gain. In contrast, when comparing female *Trpm5* ^*-/-*^ mice fed the HFD to weight matched younger wild type mice they exhibited similar fasting insulin and glucose levels and HOMA-IR index (Table 3, Fig 5A and 5C).

**Table 3:** Body weight, fasting insulin and glucose of female wild type (WT) and *Trpm5* ^*-/-*^ (KO) mice at different time points when fed HFD or cafeteria diet.

	Cafeteria diet		HFD	
	WT (n = 8)	KO (n = 5)	WT (n = 13)	KO (n = 24)
**w. 0 BW (g)**	20.9 ± 0.5	21.3 ± 0.5	22.4 ± 0.4	21.1 ± 0.2
**w. 0 Fasting Glucose (mM)**	8.4 ± 0.3	8.9 ± 0.4	9.4 ± 0.2	9.4 ± 0.3
**w. 0 Fasting Insulin (ng/ml)**	0.38 ± 0.02	0.36 ± 0.04	0.68 ± 0.13	0.50 ± 0.09
**w. 0 HOMA-IR**	3.9 ± 0.2	4.1 ± 0.6	7.8 ± 1.4	6.4 ± 1.3
**w. 20 BW (g)**	*50*.*6 ± 1*.*4*	*45*.*4 ± 2*.*0*	**47.4 ± 0.9**	*37*.*9 ± 1*.*1* ***
**w. 20 Fasting Glucose (mM)**			**9.8 ± 0.3**	9.6 ± 0.1
**w. 20 Fasting Insulin (ng/ml)**			**0.90 ± 0.07**	0.56 ± 0.05***
**w. 20 HOMA-IR**			**11.1 ± 1.1**	6.6 ± 0.5***
**w. 21 BW (g)**	**50.0 ± 1.4**	*44*.*4 ± 2*.*1* *	*46*.*7 ± 1*.*0g*	*38*.*3 ± 1*.*1g*
**w. 21 Fasting Glucose (mM)**	**12.6 ± 1.0**	*9*.*9 ± 0*.*8* ^*0*.*08*^		
**w. 21 Fasting Insulin (ng/ml)**	**3.4 ± 0.4**	*1*.*2 ± 0*.*3* **		
**w. 21 HOMA-IR**	**50.4 ± 2.9**	*15*.*4 ± 4*.*9* ***		
**w. 27 BW (g)**	**54.5 ± 1.5**	**49.5 ± 2.3** ^**0.08**^	*51*.*8 ± 1*.*0*	*41*.*3 ± 1*.*5g*
**w. 27 Fasting Glucose (mM)**	**11.2 ± 0.6**	**9.7 ± 0.6** ^**0.1;**^ ^**a**^		
**w. 27 Fasting Insulin (ng/ml)**	**6.1 ± 1.2**	**2.0 ± 0.3** * ^;^ ^**a**^		
**w. 27 HOMA-IR**	**83.8 ± 14.8**	**23.8 ± 3.8** ** ^;^ ^**c**^		
**w. 37 BW (g)**	*61*.*5 ± 1*.*8g*	*53*.*6 ± 3*.*5g*	*55*.*8 ± 1*.*0*	**44.4 ± 1.8** ***
**w. 37 Fasting Glucose (mM)**			*10*.*2 ± 0*.*3*	**9.8 ± 0.4**
**w. 37 Fasting Insulin (ng/ml)**			*2*.*5 ± 0*.*3*	**1.1 ± 0.2** ***
**w. 37 HOMA-IR**			*31*.*8 ± 3*.*5*	**13.5 ± 2.4** ***
**w. 39 BW (g)**	*63*.*0 ± 1*.*7*	**52.8 ± 3.1** **	*57*.*1 ± 1*.*0g*	*45*.*1 ± 1*.*8g*
**w. 39 Fasting Glucose (mM)**	*10*.*9 ± 0*.*4*	**9.4 ± 0.5** * ^;^ ^**a**^		
**w. 39 Fasting Insulin (ng/ml)**	*6*.*4 ± 0*.*7*	**2.8 ± 1.1** * ^**; 0.07**^		
**w. 39 HOMA-IR**	*86*.*3 ± 9*.*7*	**31.7 ± 10.7** ** ^;^ ^**a**^		

The mice were fed the high caloric diets from the age of 12 weeks (w. 0). The week notation then corresponds to number of weeks being on respective diets. To determine whether effects on glucose and insulin homeostasis were directly dependent of the weight gain, the later time points chosen for the oral glucose tolerance test (OGTT) was when the *Trpm5* ^*-/-*^ mice had reached the same bodyweight as the wild type mice at the preceding OGTT. Arrows indicates when a similar body weight was reached. Data are presented as mean ± SEM.

*<0.05

**p<0.01

***p<0.001 *Trpm5* ^*-/-*^ vs corresponding wild type control at the same age within respective diet (students t-test).

^a^p<0.05

^c^p<0.001 *Trpm5* ^*-/-*^ vs corresponding wild type control at a similar body weight within respective diet (students t-test).

When comparing the effects of the different diets within the same genetic background and at similar body weights, the HOMA-IR index was increased 2.6 times in female wild type mice fed the cafeteria diet compared to when fed HFD (83.8 ± 14.8 week 27 vs 31.8 ± 3.5 week 37, p<0.001). Whereas, for the *Trpm5* ^*-/-*^ mice the HOMA-IR index was similar when fed the cafeteria diet compared to when fed HFD (15.4 ± 4.9 week 21 vs 13.5 ± 2.4 week 37, p<0.05,) (Fig 5A). This implies that the *Trpm5* ^*-/-*^ mice were resistant to the high sugar cafeteria diet induced glucose intolerance.

**Fig 6:**
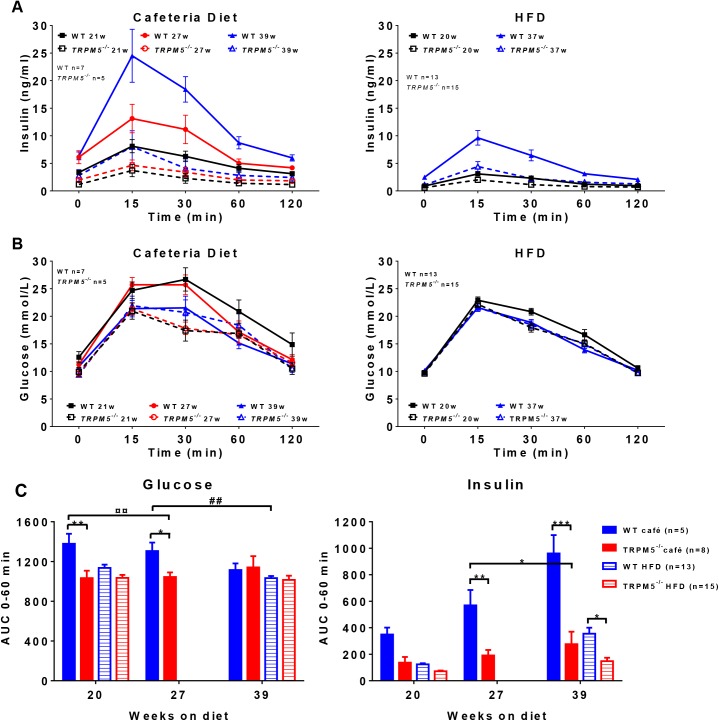
Effects of diet on glucose and insulin responses after an oral glucose tolerance test in female *Trpm5* ^*-/-*^ and wild type mice. At different time points (weeks) after start on respective diet an oral glucose tolerance test was performed after a 4h fasting and (A) insulin and (B) glucose levels were recorded. (C) The area under the curve (AUC) was calculated for the first 60 min of the glucose and insulin response curves. Data are presented as mean ± SEM, *p<0.05, **p<0.01, ***p<0.001, *Trpm5* ^*-/-*^ vs wild type at corresponding age and diet, ^¤^p<0.05, ^¤¤¤^p<0.001,*Trpm5* ^*-/-*^ vs wild type mice at corresponding bodyweight and diet, ^##^p<0.01 cafeteria diet vs HFD for respective genotype (ANOVA with Bonferroni post-hoc test).

When challenged with an oral glucose load (oral glucose tolerance tests, OGTT), female *Trpm5* ^*-/-*^ mice showed lower insulin response at all occasions and lower glucose levels after 20 and 27 weeks when fed either cafeteria diet or HFD compared to age matched wild type mice (Fig 6A and 6B). Interestingly, when comparing weight matched wild type mice on the different diets (cafeteria diet vs HFD), the insulin levels tended to be increased and the glucose levels were significantly elevated when wild type mice were fed cafeteria diet compared to HFD (insulin AUC_0-60 min_ 569 ± 116 week 27 vs 355 ± 46 week 37, and glucose AUC_0-60 min_ 1305 ± 85 week 27 vs 1033 ± 21 week 37 respectively, p<0.01). Whereas, weight matched *Trpm5* ^***-/-***^ mice showed no difference in either glucose or insulin levels when fed cafeteria diet compared to when fed HFD (Fig 6C). Thus *Trpm5* ^***-/-***^ mice seems to be resistant to the pre-diabetic condition induced by the sugar enriched cafeteria diet in wild type mice.

### *Trpm5* ^*-/-*^ mice lack preference for fat/sugar-rich chocolate food, but retain energy content driven intake

**Fig 7:**
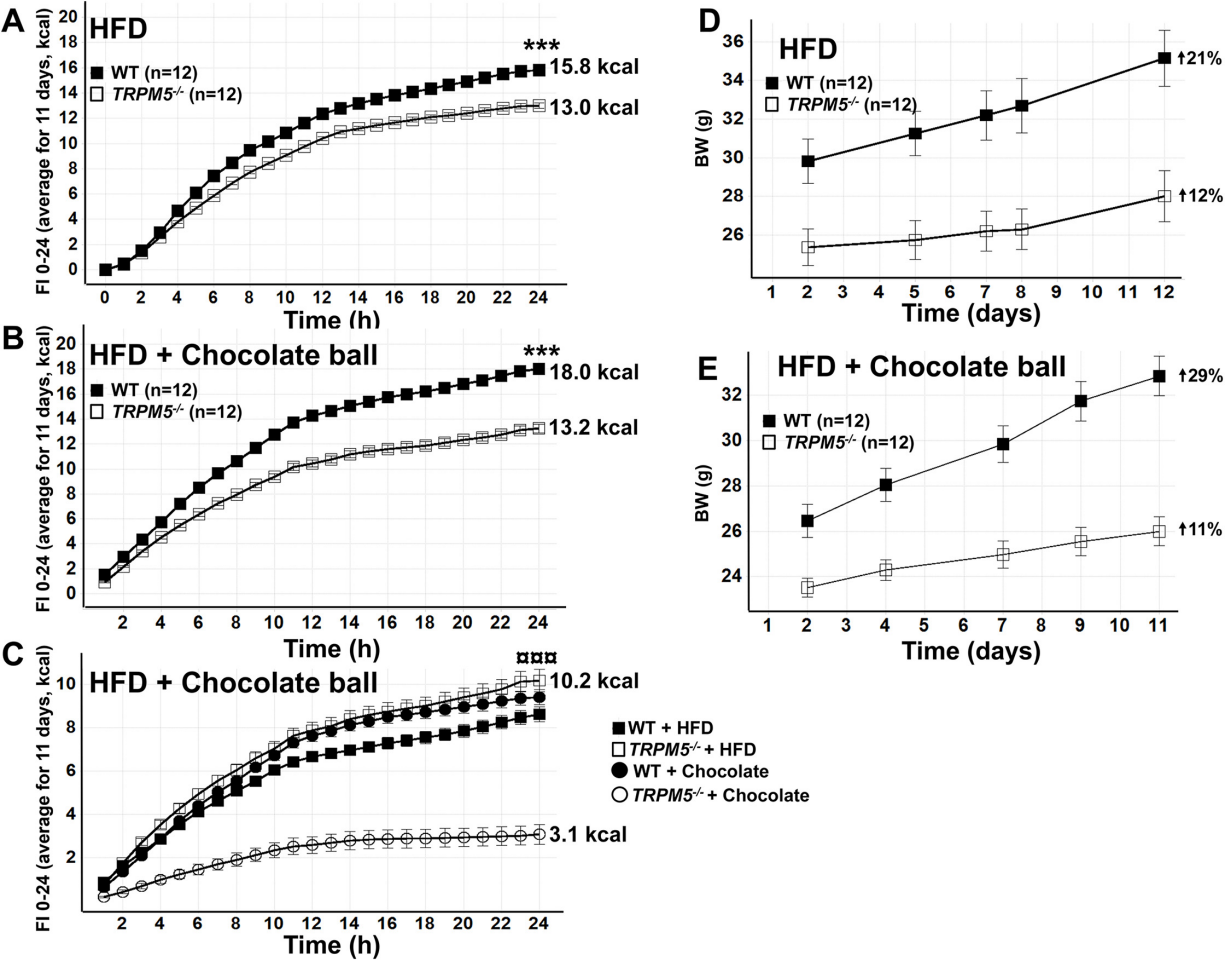
Effects of diets on caloric intake and preference of respective diet in female *Trpm5* ^*-/-*^ and wild type mice. Total caloric intake of HFD alone (A), of HFD and a chocolate ball when offered together (B) and of HFD and chocolate ball respectively when offered together (C). Body weight gain when offered HFD alone (D) or when offered HFD and a chocolate ball together (E). Data are presented as mean ± SEM, ***p<0.001, total caloric intake *Trpm5* ^*-/-*^ vs wild type mice, ¤¤¤ p<0.001 caloric intake from HFD vs chocolate ball for *Trpm5* ^*-/-*^ mice (Students t-test).

Female *Trpm5* ^***-/-***^ mice consumed significantly fewer calories compared to wild type mice over 24h when fed either HFD only or HFD in combination with the chocolate ball (p<0.001, Fig 7A and 7B). With access to HFD only or both HFD and the chocolate ball in separate food hoppers, *Trpm5* ^***-/-***^ mice consumed the same amount of total calories (13 kcal/ 24 h/ mouse) whilst wild type mice increased their total consumption (from 16 to 18 kcal/ 24 h/ mouse) when offered both diets (Fig 7A and 7B). With access to both food types, *Trpm5* ^***-/-***^ mice consumed a similar amount of HFD (10 kcal/ 24 h/ mouse vs 9 kcal/ 24 h/ mouse), but significantly less of the chocolate ball (3 kcal/ 24 h/ mouse vs 9 kcal/ 24 h/ mouse) compared to wild type mice (Fig 7C). On both diets, the reduced food intake was reflected in decreased body weight gain in *Trpm5* ^***-/-***^ mice compared to wild type mice (Fig 7D and 7E).

**Fig 8:**
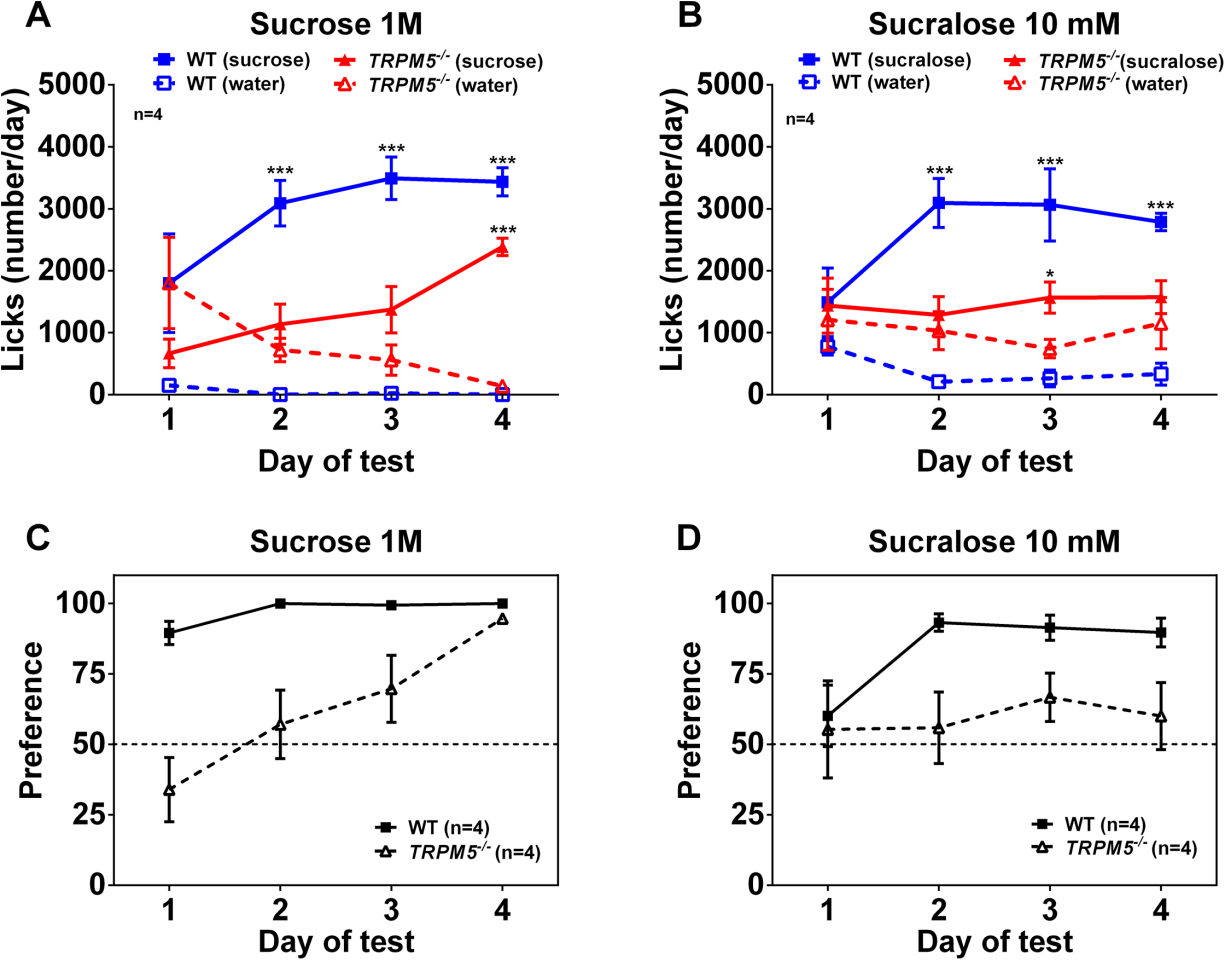
Lickometer taste preference test in male *Trpm5* ^*-/-*^ and wild type mice. Preferences of 1M sucrose or 10 mM sucralose over water were followed during 4 consecutive days. Average daily number of licks of water and 1M sucrose (A) or 1 mM sucralose (B) for *Trpm5* ^*-/-*^ and wild type mice were calculated. A preference score (percent licks on the test solution of the total number of licks) were calculated for 1M sucrose (C) and 10 mM sucralose (D). The dashed line (50% preference) indicates when the mouse consume equal amount of water and liquid tastant. Data are presented as mean ± SEM, n = 4, *** p<0.001, water vs sweetener within the same genotype (Students t-test).

To further investigate the ability of the mice to sense and show preference for the nutrient content, a lickometer taste preference test was performed. As reported above, male *Trpm5* ^***-/-***^ mice did not show an acute preference for either sucrose or the artificial sweetener sucralose as observed with wild type mice. However, after 3 days in the nutrient preference test, *Trpm5* ^***-/-***^ mice developed a preference for sucrose but not sucralose (Fig 8).

### *Trpm5* ablation did not affect energy expenditure or spontaneous locomotor activity

**Fig 9:**
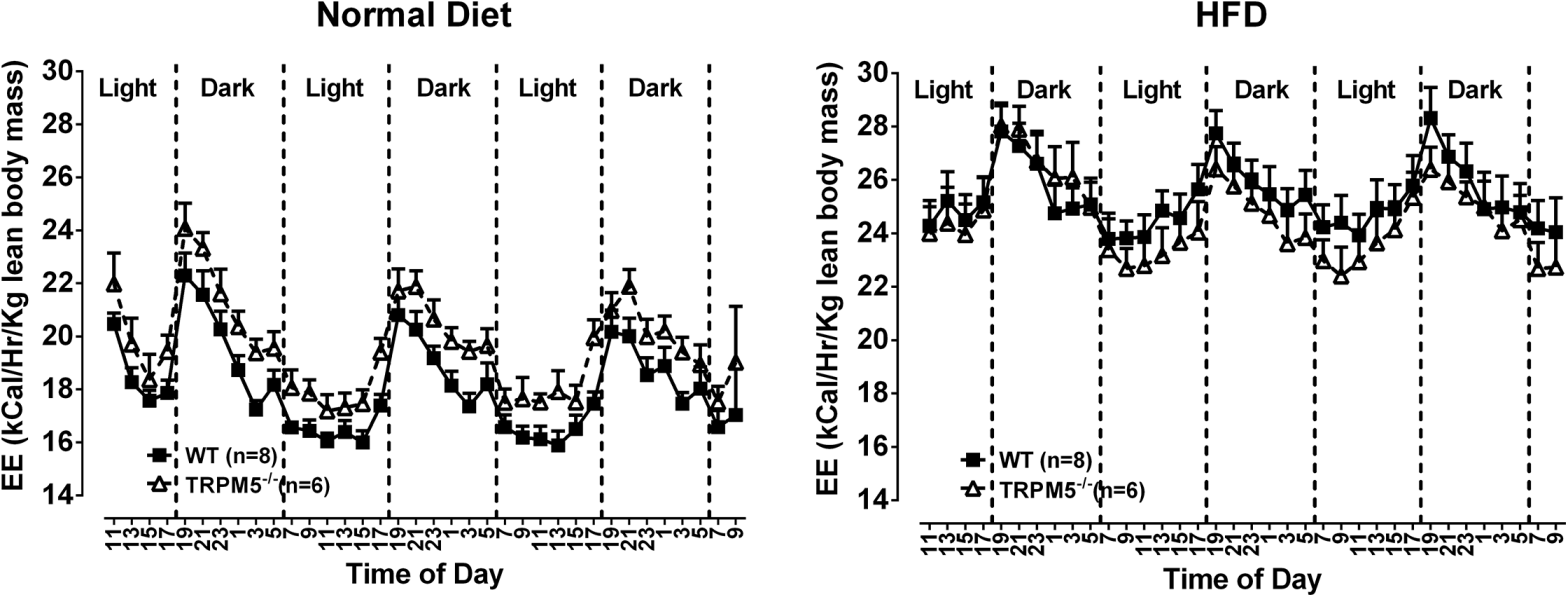
Energy expenditure test in male *Trpm5* ^*-/-*^ and wild type mice. Time course of energy expenditure calculated on lean body mass during 72 hours before (A) and after (B) being on HFD for 10 weeks, shown on the X-axis are the actual clock hours. Data are presented as mean ± SEM.

To further investigate the mechanism for the reduced weight gain in *Trpm5* ^***-/-***^ mice, energy expenditure and locomotor activity were assessed in male *Trpm5* ^***-/-***^ and wild type mice before and after 10 weeks of HFD. However, there were no detectable differences in either energy expenditure (Fig 9) or locomotor activity (data not shown) between *Trpm5* ^***-/-***^ mice and wild type mice before starting on the HFD or after 10 weeks of HFD.

## Discussion

In this study we show that *Trpm5* ^*-/-*^ mice gained significantly less body weight and fat mass on both palatable carbohydrate rich cafeteria diet and 60% HFD and were more glucose tolerant compared to wild type mice, which after cafeteria diet induced obesity was largely independent of body weight. We also demonstrated that palatable sugar and fat containing diet induced overeating in wild type mice, which was prevented in *Trpm5* ^*-/-*^, who consumed the same amount of energy from a HFD and a combined HFD and sugar rich diet. Thus, the lack of sweet taste preference observed in *Trpm5* ^*-/-*^ mice might explain why these mice become less obese and glucose intolerant on cafeteria diet. The results also point to sweet taste induced overeating as the cause for the increased energy intake and glucose intolerance seen for wild type mice on a combined sugar and high fat rich diet compared to when on a HFD only.

Consistent with earlier findings [22,23] we showed that the *Trpm5* ^*-/-*^ mice, in contrast to wild type littermates, did not have any preference for either sucrose or the artificial sweetener sucralose in an acute two bottle lickometer test, confirming that the *Trpm5* ^*-/-*^ mice used in the studies were functionally deficient in their sweet taste perception. In this context it is important to consider that several different *Trpm5* ^*-/-*^ strains are used in the literature. The *Trpm5* gene is large, and the existing knockout constructs are designed in different ways, possibly resulting in slightly different phenotypes both when it comes to taste deficiency and metabolic parameters.

The mice were started on diet at the age of 12 weeks, since we wanted to explore if there was any apparent difference between wild type and *Trpm5* ^*-/-*^ mice before starting the high caloric diet studies. This could not be found, *Trpm5* ^*-/-*^ and wild type mice had similar body weights and they also showed similar glucose and insulin levels before (fasting) and after an oral glucose tolerance tests (OGTT) before starting on respective diets. When fed the cafeteria diet, the *Trpm5* ^*-/-*^ mice gained significantly less body weight, total fat and lean body mass compared to wild type mice after 40 weeks on the diet, which is consistent with prior results in short term studies [36]. The lower adiposity of the *Trpm5* ^*-/-*^ mice is most likely due to the reduced weight gain, since there is no difference in either % total body fat or % ovarian fat compared to wild type littermates. The reduced body weight gain in *Trpm5* ^*-/-*^ mice could potentially be due to their impaired taste response demonstrated in the current study and by others [22,23], thereby reducing their intake of the palatable cafeteria diet. In the follow up high caloric diet study, *Trpm5* ^*-/-*^ and wild type mice were thus given a defined 60% HFD only (D12492). Interestingly, the reduced body weight gain in *Trpm5* ^*-/-*^ mice compared to wild type mice were even more pronounced when fed the HFD compared to the cafeteria diet. Therefore, experiments were performed to investigate if differences in food intake could explain the differences in weight gain. The food intake was automatically recorded for single housed mice, being fed HFD only or a combination of HFD in one food hopper and a sugar-rich chocolate ball in the other food hopper. On HFD only, *Trpm5* ^*-/-*^ mice consumed significantly fewer calories compared to wild type mice. Interestingly, when given HFD plus the chocolate ball together, *Trpm5* ^*-/-*^ mice still consumed the same amount of calories as they did on HFD only, while wild type mice significantly increased their total intake of calories, mainly due to excessive consumption of the palatable chocolate ball. The reduced consumption of chocolate in *Trpm5* ^*-/-*^ mice compared to wild type mice is presumably related to their inability to recognise sweet taste and may also reflect that HFD have more nutritional value compared to chocolate. This is also reflected in the eating pattern of the two diets, where fewer and larger meals of HFD compared to chocolate ball were registered (data not shown). We thus propose that food intake seems to mainly be driven by the total energy content of the food in *Trpm5* ^*-/-*^ mice and that the excess consumption of chocolate in wild type mice is most likely due to their hedonic drive for sweet taste, which is lost in *Trpm5* ^*-/-*^ mice. There are several reports describing that both oral (food palatability) and post-oral (nutritional) value play central roles in nutrient intake (reviewed in [38,39]). In accordance to previous studies [36] we also demonstrated that *Trpm5* ^*-/-*^ mice after 3 days developed a preference for liquid sucrose but not sucralose, demonstrating the preference for nutritional value detached from taste. However, the development of preference for sucrose but not the chocolate ball is somewhat contradictory and further research is needed to explore differences in hedonic and nutritional value between solid sweet food and sweet liquids. It should be noted that the *Trpm5* ^*-/-*^ mice gained less weight on both the cafeteria and the HFD in the long term high caloric diet studies. As we have discussed, the higher consumption of cafeteria diet by the wild type mice could be due to sweet taste, not recognised by the *Trpm5* ^*-/-*^ mice. The reason for the lower body weight gain of *Trpm5* ^*-/-*^ mice on HFD might be due to the involvement of TRPM5 in fat and non-sweet tasting carbohydrate preference ([32,33]).

Another explanation for the reduced weight gain in *Trpm5* ^***-/-***^ mice could be increased energy expenditure and/or locomotor activity. However, we were not able to detect any differences in either energy expenditure or locomotor activity between male *Trpm5* ^***-/-***^ and wild type mice, neither before starting on HFD or after being on HFD for 12 weeks. This is consistent with previous data [40].

It is well established that excessive body weight is associated with impaired glucose control and increased insulin secretion. Thus, the improved glucose control in *Trpm5* ^***-/-***^ mice, when compared at the same age after >20 weeks on the high caloric diets (both cafeteria diet and HFD), was most likely due to the reduced body weight gain of *Trpm5* ^***-/-***^ mice compared to wild type mice. However, when comparing cafeteria diet fed *Trpm5* ^*-/-*^ mice with weight matched younger wild type mice, *Trpm5* ^***-/-***^ mice still exhibited a significantly improved HOMA-IR index, as well as reduced insulin secretion and improved glucose clearance after an oral glucose challenge. This is contradictory with previous data, where a different strain of *Trpm5* ^***-/-***^ mice have been shown to have impaired glucose tolerance, due to reduced glucose induced insulin release from β-cells [29,30]. However, these experiments were performed on 10–14 weeks old mice on regular mouse show, which is comparable to our mice when they were started on high caloric diet. At this age (12 weeks) we were not able to detect any difference between *Trpm5* ^***-/-***^ and wild type mice, neither on fasting glucose and insulin levels nor after a glucose challenge. However, we did only fast our mice for 4h, whereas Colsoul *et al*. [29] fasted their mice over night, a very long fasting time for a mouse, which could explain the discrepancy between the studies. The defective insulin secretion from pancreatic β-cells in *Trpm5* ^***-/-***^ mice [29] could explain the lower insulin secretion, but is contradictory to the improved glucose control. However, in this context it is important to note that the *Trpm5* ^***-/-***^ *construct used by* Colsoul *et al*. [29] is not the same as the Deltagen *Trpm5* ^***-/-***^ strain that is used in this paper. The *Trpm5* gene is large, and the existing knockout constructs are designed in different ways, possibly resulting in slightly different phenotypes.

In conclusion, the main findings of this study are the improved glucose tolerance and HOMA:IR, which indicates an improved insulin sensitivity in *Trpm5* ^***-/-***^ mice compared to wild type mice on cafeteria diet, which is clearly body weight independent, and the observed overeating of a combined sugar and fat rich diet (chocolate ball) by the wild type mice. Together these observations suggest an important role for TRPM5 in diet induced glucose intolerance. The lack of sweet taste preference observed in *Trpm5* ^***-/-***^ mice might explain why these mice become less obese and less glucose intolerant on cafeteria diet, and together with the results obtained after the long term cafeteria diet, indicates that sweet taste induced overeating may be the cause for the increased energy intake and insulin resistance seen for wild type mice on sugar rich diet compared to HFD. Glucose clamp studies and experiments with pair feeding of *Trpm5* ^***-/-***^ and wild type mice would be interesting follow up studies to further explore this.
